# Gender-Associated Genes in Filarial Nematodes Are Important for Reproduction and Potential Intervention Targets

**DOI:** 10.1371/journal.pntd.0000947

**Published:** 2011-01-25

**Authors:** Ben-Wen Li, Amy C. Rush, Dao-Jun Jiang, Makedonka Mitreva, Sahar Abubucker, Gary J. Weil

**Affiliations:** 1 Infectious Diseases Division, Washington University School of Medicine, St. Louis, Missouri, United States of America; 2 Department of Genetics, The Genome Center, Washington University School of Medicine, St. Louis, Missouri, United States of America; McGill University, Canada

## Abstract

**Background:**

A better understanding of reproductive processes in parasitic nematodes may lead to development of new anthelmintics and control strategies for combating disabling and disfiguring neglected tropical diseases such as lymphatic filariasis and onchocerciasis. Transcriptomatic analysis has provided important new insights into mechanisms of reproduction and development in other invertebrates. We have performed the first genome-wide analysis of gender-associated (GA) gene expression in a filarial nematode to improve understanding of key reproductive processes in these parasites.

**Methodology/Principal Findings:**

The Version 2 Filarial Microarray with 18,104 elements representing ∼85% of the filarial genome was used to identify GA gene transcripts in adult *Brugia malayi* worms. Approximately 19% of 14,293 genes were identified as GA genes. Many GA genes have potential *Caenorhabditis elegans* homologues annotated as germline-, oogenesis-, spermatogenesis-, and early embryogenesis- enriched. The potential *C. elegans* homologues of the filarial GA genes have a higher frequency of severe RNAi phenotypes (such as lethal and sterility) than other *C. elegans* genes. Molecular functions and biological processes associated with GA genes were gender-segregated. Peptidase, ligase, transferase, regulator activity for kinase and transcription, and rRNA and lipid binding were associated with female GA genes. In contrast, catalytic activity from kinase, ATP, and carbohydrate binding were associated with male GA genes. Cell cycle, transcription, translation, and biological regulation were increased in females, whereas metabolic processes of phosphate and carbohydrate metabolism, energy generation, and cell communication were increased in males. Significantly enriched pathways in females were associated with cell growth and protein synthesis, whereas metabolic pathways such as pentose phosphate and energy production pathways were enriched in males. There were also striking gender differences in environmental information processing and cell communication pathways. Many proteins encoded by GA genes are secreted by *Brugia malayi*, and these encode immunomodulatory molecules such as antioxidants and host cytokine mimics. Expression of many GA genes has been recently reported to be suppressed by tetracycline, which blocks reproduction in female *Brugia malayi*. Our localization of GA transcripts in filarial reproductive organs supports the hypothesis that these genes encode proteins involved in reproduction.

**Conclusions/Significance:**

Genome-wide expression profiling coupled with a robust bioinformatics analysis has greatly expanded our understanding of the molecular biology of reproduction in filarial nematodes. This study has highlighted key molecules and pathways associated with reproductive and other biological processes and identified numerous potential candidates for rational drug design to target reproductive processes.

## Author Summary

Lymphatic filariasis is a neglected tropical disease that is caused by thread-like parasitic worms that live and reproduce in lymphatic vessels of the human host. There are no vaccines to prevent filariasis, and available drugs are not effective against all stages of the parasite. In addition, recent reports suggest that the filarial nematodes may be developing resistance to key medications. Therefore, there is an urgent need to identify new drug targets in filarial worms. The purpose of this study was to perform a genome-wide analysis of gender-associated gene transcription to improve understanding of key reproductive processes in filarial nematodes. Our results indicate that thousands of genes are differentially expressed in male and female adult worms. Many of those genes are involved in specific reproductive processes such as embryogenesis and spermatogenesis. In addition, expression of some of those genes is suppressed by tetracycline, a drug that leads to sterilization of adult female worms in many filarial species. Thus, gender-associated genes represent priority targets for design of vaccines and drugs that interfere with reproduction of filarial nematodes. Additional work with this type of integrated systems biology approach should lead to important new tools for controlling filarial diseases.

## Introduction

*Wuchereria bancrofti, Brugia malayi* and *Brugia timori* are the causative agents of lymphatic filariasis (LF), which is a disabling and disfiguring parasitic disease. An estimated 120 million people are infected, and more than 1.3 billion people are at risk for this disease in subtropical and tropical regions of the world [1]. Current treatments rely on a limited number of drugs, namely diethycarbamazine, albendazole and ivermectin. Although these treatments are partially effective, the molecular effects of these drugs on filarial nematodes are not completely understood [2]. In addition, these drugs are not effective against all parasite stages, and recent reports suggest that the parasites may be developing resistance to treatment [3], [4]. Therefore, the search for new drug targets and effective vaccine candidates is an important priority. Improved understanding of genes that are critically important for embryo development and reproduction may lead to new ways to combat these parasitic diseases [5].

Profiling of gender-associated gene expression has proven to be very useful for elucidating molecular mechanisms of reproduction and for identifying gender-specific or enriched genes involved in reproductive processes [6], [ 7], [ 8], [ and 9]. This approach has led to new interventions against arthropods. For example, a vaccine that targets egg formation that came out of studies of gender-specific gene expression has reduced egg production in *Boophilus microplus*, an important tick parasite of livestock [10]. Female-specific lethality has been achieved by an engineered autocidal genetic system in insects [11]. Thus, improved understanding of the molecular biology of reproduction in parasitic nematodes may explain the differential activity of certain anthelmintics in male and female worms [12], [13].

Transcriptomic approaches have been useful for studying transcription profiles associated with drug treatment [14], environmental stress [15], [16], host immune responses [17] and reproduction [7], [8]. This approach has been used also to identify genes that are critical for reproductive processes such as embryogenesis in *C. elegans* [18] and sexual maturation and oviposition in *Schistosoma* [19]. Filarial nematodes are dioecious, and they exhibit marked sexual dimorphism. We have previously identified genes with gender-associated expression in *B. malayi* adult worms based on cDNA oligonucleotide array analysis with the version 1 Filarial Microarray (BmV1 array) [8]. That study provided the first broad view of gender-associated gene expression in filarial nematode. Together with comparative analyses of data from the free-living nematode *C. elegans*, molecular investigations are beginning to provide insight into processes involved in reproduction and development in filarial nematodes [8], [20]. Focusing attention on products and pathways that have biological roles in the formation of sexually mature worms and eggs has improved understanding of the biology of reproduction and identified genes that influence immunopathology or immunomodulation [8], [20], [21], [22]; some of these genes may represent novel chemotherapeutic targets [23].

The present study has greatly expanded our previous analysis of gender-associated gene expression, which was limited to transcripts from a limited number of *Brugia* EST's (3,569). The current study represents a global gene expression analysis with version 2 Filarial Microarray (BmV2 array), which includes elements that represents approximately 85% of the *B. malayi* genome [24]. We have also performed a detailed systematic functional analysis of gender-associated transcripts to identify genes and pathways associated with reproductive activities and other developmental processes. This comprehensive data set provides a foundation of information that will facilitate future hypothesis-directed drug and vaccine development.

## Materials and Methods

### Ethics statement

The animal work was carried out under protocols #20050377 and 20090045 approved by the Animal Studies Committee of Washington University School of Medicine at St Louis, Missouri, USA.

### Parasite materials

Adult *B. malayi* worms were isolated from the peritoneal cavity of infected jirds (*Meriones unguiculatus*) obtained from the NIAID Filariasis Research Reagent Resource Center (FR3) (University of Georgia, Athens, GA). Mature male and female worms were carefully separated by size and morphology; broken worms were discarded. The worms were carefully washed in PBS and immediately frozen at −80°C.

### RNA isolation and probe preparation

Total RNA was prepared from 30 mature adult worms per batch using TRIzol (GibcoBRL, Life Technologies) as previously described [8]. cDNA was synthesized from 5–7 ug female or male total RNA using 3DNA capture sequence primers (3DNA Array 350 Detection system, Genisphere, Hatfield, PA) and SuperScript II Reverse Transcriptase (Gibco BRL, Gaithersburg, MD) for each probe according to standard protocols. cDNA was concentrated using a Microcon YM-100 filter (Millipore) and either used immediately or stored at −80°C. cDNA synthesized from two different batches of male and female RNA samples that were independently prepared were used as biological replicates. A two-step protocol was used for hybridization (3DNA Array 350 Detection system, Genisphere, Hatfield, PA) as previously described [8]. Each experiment consisted of a pair-wise competitive hybridization of cDNA samples (male/female) with reciprocal dye-flip replicates. Because biological replicates and dye-flip replicates were tested, a total of four DNA microarrays were used for each comparison of two types of cDNA. Eight hybridizations were performed for each element on the array, as all probes are present in duplicate on the array.

### Microarray fabrication

The BmV2 array contains 18,104 elements derived from *B. malayi* (15,412), *Onchocerca volvulus* (1,016), *Wuchereria bancrofti* (872) and *Wolbachi*a (*wBm*, 804 genomic elements. All information regarding the BmV2 array including oligo name, sequence and source corresponding to the *B. malayi* genome (Bm1_nomenclature) and *B. malayi* peptide models (XXXXX.m00YY nomenclature) and *B. malayi* gene index (ESTs, TC nomenclature) and full length consensus sequences are available from http://www.filariasiscenter.org/brugia-malayi-genomics-and-bioinformatics-resources/. The features of the BmV2 array have been previously described [16]. The oligonucleotides (50 nM in 3× SSC with 0.75 M betaine) were printed in duplicate on MWG Epoxy slides (MWG Bioteche Inc, High Point, NC) by a locally constructed linear servo-arrayer (after the DeRisi model, http://derisilab.ucsf.edu).

### Data processing and analysis

Slide scanning and image analysis were performed as described previously [16]. Briefly, slides were scanned immediately after hybridization on a ScanArray Express HT Scanner (Perkin Elmer, Boston, MA) to detect Cy3 and Cy5 fluorescence at 543 and 633 nm, respectively. The scanner produces green Cy3 and red Cy5 16-bit TIFF image files and extracts intensities from the scanner images for both dyes. Signal values were backgrounds subtracted and Lowess [25], [26] normalized by using GeneSpring version 6.1 software (Silicon Genetics, Redwood City, CA). Twenty percent of the data was used to calculate the Lowess fit at each point. Oligonucleotide elements that received “present” calls in all four microarrays and displayed >700 or >127 (high or low PMT settings, respectively) in 2 of 4 channels for either the Cy3 or Cy5 were identified, all others were excluded from the analysis. The log_2_ ratio of median dye intensities for each remaining element was averaged across all four microarrays. Genes with equal to or greater than two-fold differences in expression and a confidence level of 99% (*P*<0.01, Student's *t*-test) in pair-wise comparison were considered to be differentially expressed. A complete list of elements for the array with oligonucleotide sequences and hybridization data are available online at http://nematode.net/Microarray/index.php.

### Annotation and functional assignment

For functional annotation of the transcripts, the program Blastx was used to compare nucleotide sequences to various databases [27]. The full length consensus sequences corresponding to gender associated elements (http://www.filariasiscenter.org/brugia-malayi-genomics-and-bioinformatics-resources/) were queried against the non-redundant (NR) protein database of the National Center for Biotechnology Information (NCBI) and a *C. elegans* database (Wormpep 195). The best potential homologues were reported with a probability of 1e-05 or better. To identify cases where the potential filarial homologues in *C. elegans* have been surveyed for knockout phenotypes using RNA interference (RNAi) [28], [ 29], [ and 30], the available RNAi information was extracted using in-house developed perl scripts.

InterProScan v13.1 was used to assign gender-associated element consensus sequences to known InterPro domains, with subsequent mapping into the Gene Ontology (GO) hierarchy [31], [32]. Statistically significant enrichment of GO categories by gender (e.g. filarial male-enriched) over the background (complete BmV2 array gene set) was calculated using a hypergeometric test with the *P*-value cutoff of p<0.01. Less informative ontology terms, including those at level 4 or higher for Biological Process or Molecular Function, and those at level 2 or higher for Cellular Component, were removed from the enrichment list.

Gender-associated elements were assigned by enzyme commission number to metabolic pathways using the KEGG database. An e-value cut-off of 1.0e-10 reported by WU-BLASTP against Genes Database Release 39.0 from Kyoto Encyclopedia of Genes and Genomes (KEGG) was used for pathway mapping; the top match and all of the matches within a range of 30% of the top BLAST score that met the cut-off were accepted as valid KEGG associations [33], [34]. A hypergeometric analysis (measuring the coverage of KEGG Orthology (KO) gene groups for each KEGG pathway compared to the complete gene set on BmV2 array) was implemented to identify enriched pathways for each gene set [35].

### Validation of gender-associated transcription by real-time qRT-PCR

Reverse transcription real-time PCR (qRT-PCR) was performed to independently assess gene expression by gender for 20 genes with GA expression by microarray as previously described [36]. The quality of qRT-PCR reactions was verified by amplification efficiency and melting curves analysis as described previously [36]. Three genes (Table S1) with consistent expression in male and female worms were chosen as endogenous controls. NADH dehydrogenase was used in previous paper and two genes (ubiquitin-like and splicing factor) are newly validated genes for endogenous controls which are better (more consistent expression in female and male worms) than the genes used in our previous study (Actin and Histone).The geometric mean of these genes was used to normalize qRT-PCR data as recently recommended [37]. The formula used to calculate fold differences was 2 ^−ΔΔ *C*t^, where the value of ΔΔ C_t_ is the difference in ΔC_t_ values obtained with the calibrator and test sample. C_t_ values obtained with 1 ng of male and female RNA starting material were used for these calculations. The sequences of the primers used for real-time PCR are listed in S1.

### *In situ* hybridization (ISH) of gender-associated genes in *B. malayi* adult worms

Protocols used for ISH were described in detail previously [38]. Briefly, RNA probes were designed with consensus sequences for selected genes downloaded from http://www.filariasiscenter.org/brugia-malayi-genomics-and-bioinformatics-resources/. The consensus sequences were blasted against *B. malayi* genome, and gene specific regions (not highly homologous to other genes) were selected for probe design. For each gene, both sense (negative control) and anti-sense probes were synthesized. Primers were designed with Primer3 (http://frodo.wi.mit.edu/primer3/) and synthesized by Integrated DNA Technology Inc. (Coralville, IA, USA). Digoxigenin labeled RNA probes were prepared by *in vitro* transcription using linearized plasmids containing *B. malayi* sequences, and probe binding was localized with antibody to digoxigenin as previously described [38]. More than 100 *B. malayi* genes have been studied by ISH during the course of the study. Results for 19 genes have been reported previously [38]. This paper reports results for four additional genes.

## Results and Discussion

### Systematic identification and confirmation of gender-associated filarial transcripts

Results obtained in this study were compared with results previously obtained with the BmV1 array which contained elements that represented 3,569 EST's [8] in order to assess consistency between the two studies. Most of the filarial genes in the two studies had consistent gender-regulated expression. This was especially true for genes with expression ratios >4 in the prior study (74% (152/205) of female- and 68% (309/455) of male upregulated elements). Some of the genes that were differentially expressed in the prior study were not confirmed in the current study. This might be due in part to different criteria used to define differential expression in the two studies (*P*<0.01 for current study vs. *P*<0.05 in the prior study) because approximately 81% of gender-associated genes from BmV1 were found to have GA expression in the current analysis if we changed the criteria (*p*<0.05. data not shown).

Twenty genes with different degree of GA expression in the current study were randomly chosen and assessed by qRT-PCR (Table 1). Although the microarray and qRT-PCR data were qualitatively similar, differences in gene expression measured by qRT-PCR were often larger than those measured by microarray (16 of 20), and this is consistent with prior studies [8], [14].

**Table 1:** Confirmation of gender-associated gene expression by qRT-PCR.

	Fold Changes			
Model Name	Microarray	qRT-PCR	Matching Pub_Locus	Description
15238.m00008	12.8 (F)	8 (F)	Bm1_55235	C hordein, putative
15241.m00008	9.8 (F)	12 (F)	Bm1_55270	hypothetical protein
12531.m00013	9.7 (F)	55 (F)	Bm1_01105	hypothetical protein CBG02248 (PH domain)
14920.m00419	7.3 (F)	147 (F)	Bm1_27950	HMG box family protein
13377.m00041	3.9 (F)	13 (F)	Bm1_08345	hypothetical protein
14961.m05178	3.2 (F)	59 (F)	Bm1_33425	variant SH3 domain containing protein
13207.m00046	3.1 (F)	27 (F)	Bm1_05930	muscle positioning protein 4, putative
14250.m00299	2.8 (F)	24 (F)	Bm1_16705	conserved hypothetical protein
12422.m00027	2.8 (F)	51 (F)	Bm1_00215	SMAll family member (sma-9)
14386.m00052	2.4 (F)	8 (F)	Bm1_18760	conserved hypothetical protein
15119.m00057	40.4 (M)	164 (M)	Bm1_54040	hypothetical protein
14317.m00267	4.7 (M)	17 (M)	Bm1_17735	hypothetical protein
BMC03352	63 (M)	35 (M)	Bm1_09765	ubiquitin-conjugating enzyme family protein
BMC03505	189 (M)	90 (M)	Bm1_02050	UNKNOWN (score 70)
14992.m11298	111.7 (M)	12 (M)	Bm1_52575	protein kinase domain containing protein
BMC03378	64.7 (M)	147 (M)	Bm1_01265	MFP2b [*Ascaris suum*]
14971.m02850	39.1 (M)	48 (M)	Bm1_36050	collagen alpha-1(XI) chain
14773.m00936	27.1 (M)	12 (M)	Bm1_25865	pyruvate dehydrogenase E1 component alpha subunit, mitochondrial, putative
14992.m11212	14 (M)	45 (M)	Bm1_52140	MFP2b [*Ascaris suum*]
14971.m02819	13.7 (M)	103 (M)	Bm1_35895	pyruvate kinase, muscle isozyme, putative

**No Note: F = Female-associated; M = Male-associated.**

Of 18,104 elements represented on the array, 14,293 had hybridization signals above the threshold for at least one sex. Of these, 2,789 elements (19%) met our criteria for gender-associated expression including 65 elements from *Onchocerca volvulus* and 94 elements from *Wuchereria bancrofti* (Table S2). Of 2,789 gender-associated elements, 1,467 elements (53%) were female-associated and 1,322 (47%) were male-associated. Similar results with more female than male-associated genes have been reported for *C. elegans* and *H. contortus* [6], [39]. However, expression profile of female worms represents female worms, microfilaria and all of the developing stages contained in the female worm (in uteri) at the time of extraction of total RNA because we did not separate all developing stages and microfilaria from mature female worms. A sequence similarity search of the 2,789 elements vs. the NCBI non-redundant (NR) database revealed that ∼58% (1,621/2,789) of gender-associated elements had significant sequence similarity to publicly available known proteins from other species (e-05) (Table S3). A significant proportion of gender regulated elements are novel (42%). These represent interesting genes that may be essential for filarial-specific reproductive processes.

### Functional characterization of filarial gender-associated genes

Domains provide important clues to protein function, and domain family-based analysis is a powerful tool for assigning function to individual proteins [40]. Fifty percent of the female- and 47% of the male-associated elements was assigned into InterPro domains (table S4). Protein kinases, phosphatases and major sperm proteins were among the most highly represented classes for male-associated elements (Table 2). This is consistent with findings in other parasitic nematodes [41] and *C. elegans* [42]. Reinke found that almost half of the protein phosphatases are associated with spermatogenesis, and protein kinases are over-represented in *C. elegans* hermaphrodites that are producing sperms. Their abundance may reflect a high demand for regulatory proteins in sperm maturation. Prior studies have shown that these enzymes are important for regulating sperm maturation by post-translational modification of proteins [42] and in signaling cascades in oocytes following fertilization [43], [44]. In contrast, a proteinase inhibitor, chaperonin, and peptidase were highly represented in female-associated elements (Table 2).

**Table 2:** Significantly overrepresented domains in female- and male-associated genes.

In Female-associated genes		
IRP	P_value	Description
IPR013201	0.0000	Proteinase inhibitor I29, cathepsin propeptide
IPR001844	0.0000	Chaperonin Cpn60
IPR013128	0.0001	Peptidase C1A, papain
IPR008950	0.0002	GroEL-like chaperone, ATPase
IPR009056	0.0004	Cytochrome c, monohaem
IPR002415	0.0004	High mobility group-like nuclear protein
IPR002468	0.0004	Peptidase M24A, methionine aminopeptidase, subfamily 2
IPR002687	0.0004	Pre-mRNA processing ribonucleoprotein, binding region
IPR012125	0.0004	Cytochrome c/c2
IPR012976	0.0004	NOSIC

In Male-associated genes		
IPR000242	0.0000	Protein-tyrosine phosphatase, receptor/non-receptor type
IPR000535	0.0000	Major sperm protein
IPR000719	0.0000	Protein kinase, core
IPR008962	0.0000	PapD-like
IPR011009	0.0000	Protein kinase-like
IPR006186	0.0000	Serine/threonine-specific protein phosphatase and bis(5-nucleosyl)-tetraphosphatase
IPR004843	0.0000	Metallophosphoesterase
IPR002290	0.0000	Serine/threonine protein kinase
IPR000387	0.0000	Protein-tyrosine phosphatase, Tyr-specific/dual-specificity type
IPR001108	0.0000	Peptidase A22A, presenilin

Using GO we associated GA genes with the three major principles of Molecular Function, Biological Processes and Cellular Components. The associations can be viewed and searched at http://www.nematode.net/cgi-bin/amigo/go_brugia_malayi_Female/go.cgi?session_id=94343301197562991703 for the female and http://www.nematode.net/cgi-bin/amigo/go_brugia_malayi_Male/go.cgi?session_id=25276231197562889484 for the male [45]. The most significant GO terms (*P*<0.01) corresponding to Molecular Function, Biological Processes and Cellular Components associated with GA genes are listed in Table 3. The significantly enriched functional classes in female-associated genes are catalytic enzymes (including peptidase, lipase, ligase), enzyme regulator and nuclear hormone receptor in transcription regulation, and genes involved in nucleic acid binding and protein binding. Elements linked to GO terms corresponding to transcription regulator activity include 5 genes encoding nuclear hormone receptors such as nuclear hormone receptor family member nhr-19 (BMC08142) and nhr-88 (14208.m00909). A unique property of nuclear receptors which differentiates them from other classes of receptors is their ability to directly interact with and control the expression of genomic DNA. Given their important regulatory role in various biological processes, nuclear receptors have long been considered to be potential drug targets [46].

**Table 3:** Significantly enriched GO terms associated with filarial gender-associated genes.

Molecular_function			
Female GO	GO Description	Male GO	GO Description
GO:0004815	aspartate-tRNA ligase activity	GO:0016301	kinase activity
**GO:0003676**	nucleic acid binding	GO:0004672	protein kinase activity
GO:0019887	protein kinase regulator activity	GO:0004725	protein tyrosine phosphatase activity
GO:0008234	cysteine-type peptidase activity	GO:0004721	phosphoprotein phosphatase activity
GO:0019904	protein domain specific binding	GO:0042578	phosphoric ester hydrolase activity
GO:0004239	methionyl aminopeptidase activity	GO:0016791	phosphoric monoester hydrolase activity
GO:0019207	kinase regulator activity	GO:0004674	protein serine/threonine kinase activity
GO:0004221	ubiquitin thiolesterase activity	GO:0016740	transferase activity
GO:0004843	ubiquitin-specific protease activity	GO:0004713	protein-tyrosine kinase activity
GO:0019843	rRNA binding	GO:0019200	carbohydrate kinase activity
GO:0030337	DNA polymerase processivity factor activity	GO:0005524	ATP binding
GO:0043565	sequence-specific DNA binding	GO:0030554	adenyl nucleotide binding
GO:0016298	lipase activity	GO:0008061	chitin binding
GO:0030528	transcription regulator activity	GO:0001871	pattern binding
		GO:0016787	hydrolase activity
		GO:0016788	hydrolase activity, acting on ester bonds
		GO:0004396	hexokinase activity

Biological_process			
Female GO	GO Description	Male GO	GO Description
GO:0007049	cell cycle	GO:0006796	phosphate metabolic process
GO:0022402	cell cycle process	GO:0006464	protein modification
GO:0050794	regulation of cellular process	GO:0006793	phosphorus metabolic process
GO:0050789	regulation of biological process	GO:0043687	post-translational protein modification
GO:0006996	organelle organization and biogenesis	GO:0043412	biopolymer modification
GO:0016070	RNA metabolic process	GO:0006468	protein amino acid phosphorylation
GO:0031323	regulation of cellular metabolic process	GO:0006470	protein amino acid dephosphorylation
GO:0006422	aspartyl-tRNA aminoacylation	GO:0016311	dephosphorylation
GO:0065007	biological regulation	GO:0016310	phosphorylation
GO:0019222	regulation of metabolic process	GO:0030247	polysaccharide binding
GO:0006350	transcription	GO:0019321	pentose metabolic process
GO:0045449	regulation of transcription	GO:0006030	chitin metabolic process
GO:0051726	regulation of cell cycle	GO:0006044	N-acetylglucosamine metabolic process
GO:0000074	regulation of progression through cell cycle	GO:0006041	glucosamine metabolic process
GO:0006355	regulation of transcription, DNA-dependent	GO:0016052	carbohydrate catabolic process
GO:0042254	ribosome biogenesis and assembly	GO:0044275	cellular carbohydrate catabolic process
GO:0022403	cell cycle phase	GO:0006040	amino sugar metabolic process
GO:0000279	M phase	GO:0044262	cellular carbohydrate metabolic process
GO:0030705	cytoskeleton-dependent intracellular transport	GO:0015980	energy derivation by oxidation of organic compounds
GO:0007018	microtubule-based movement	GO:0046365	monosaccharide catabolic process
GO:0006351	transcription, DNA-dependent	GO:0006007	glucose catabolic process
GO:0006334	nucleosome assembly	GO:0019320	hexose catabolic process
GO:0006457	protein folding	GO:0007218	neuropeptide signaling pathway
GO:0032774	RNA biosynthetic process		

Cellular_ component			
Female GO	GO Description	Male GO	GO Description
GO:0005874	microtubule	GO:0005576	extracellular region
GO:0000786	nucleosome		
GO:0005634	nucleus		
GO:0005732	small nucleolar ribonucleoprotein complex		
GO:0043626	PCNA complex		
GO:0044427	chromosomal part		

Phosphatases, kinases and proteins involved in ATP binding were significantly enriched functional classes found in male-associated transcripts. The significantly enriched biological processes in females were cell cycle, transcription and translation of cellular processes and biological regulation. By contrast, phosphate and carbohydrate metabolic processes, generation of energy, and cell communication were significantly enriched biological processes in males. Different cellular components were linked to gender-associated transcripts. Male-associated transcripts were limited to extracellular regions, whereas female-associated transcripts were linked to intracellular regions such as microtubules and the nucleosome.

**Figure 1:**
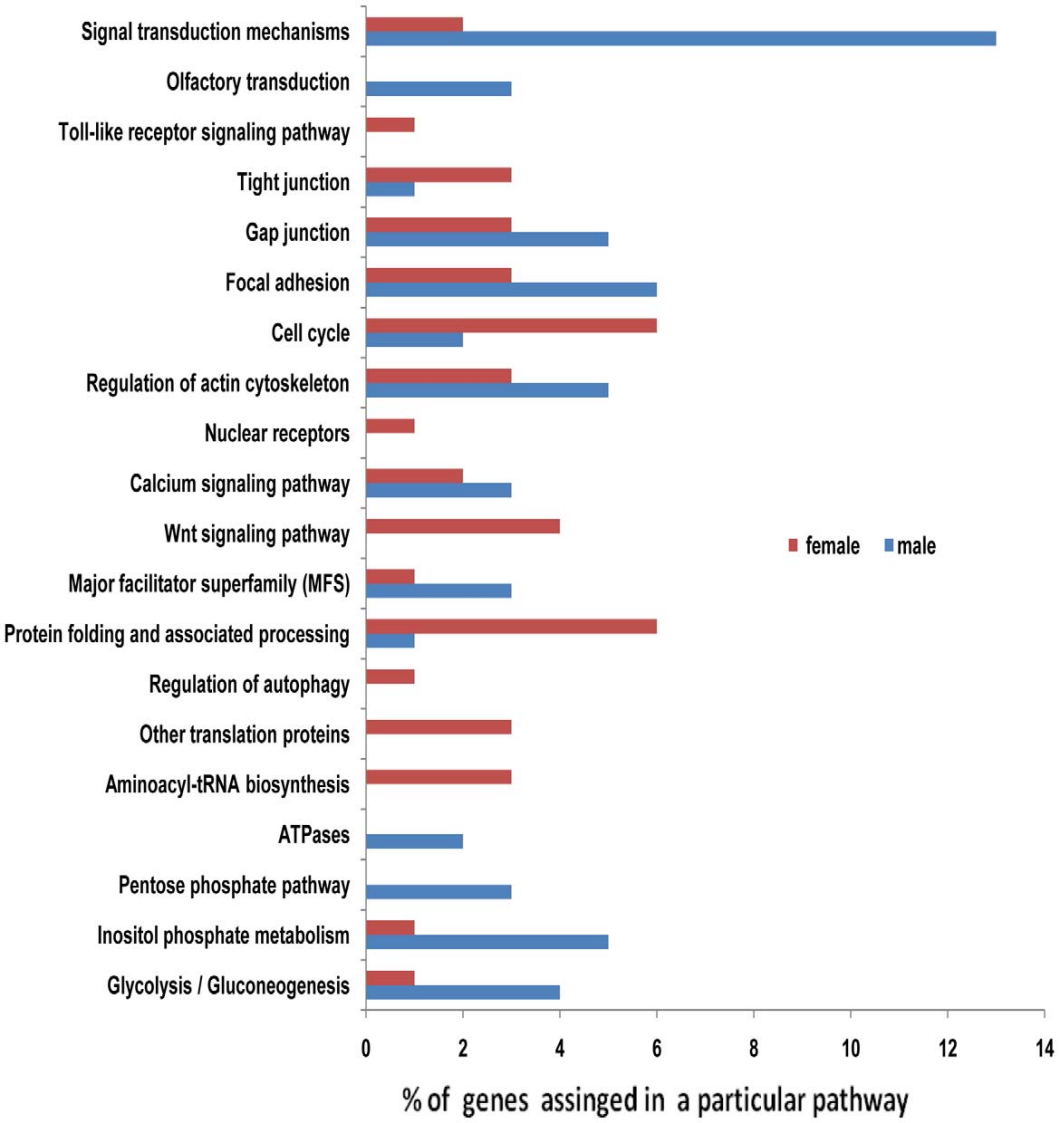
The most significant KEGG pathways were identified in gender-associated genes. KEGG pathway analyses were applied to filarial proteins encoded by filarial gender-associated genes. Values for each category are reported as percentages of functionally annotated proteins.

As an alternative categorization method, genes were assigned to pathways in the KEGG database. Enzyme commission numbers (EC) were assigned to 187 of 1,322 male-associated elements (15%) and 267 of 1,467 female-associated elements (18%) (Table S5). Cell cycle, transcription and translation related pathways such as tRNA biosynthesis emerged as significantly enriched in females, whereas metabolic pathways such as pentose phosphate, energy pathways such as oxidative phosphorylation, and cell motility in cellular processes were most significantly enriched in males (Figure 1). The difference in the use of environmental information processing pathways by males and females is striking. For example, the major facilitator superfamily (MFS) in membrane transport was significantly elevated in males. MFS is believed to function in the uptake of sugar and other metabolites [47], [48], and elevation of MFS activity may be linked to increased carbohydrate metabolism in males. In addition, the calcium signaling pathway was more abundant in males while Wnt signaling and signaling molecules pathways were elevated in females. In *C. elegans*, Wnt signaling pathways are involved in three major types of processes during development: control of asymmetric cell divisions and cell polarity, cell fate decisions and cell migration [49], and regulation of morphogenesis through regulatory myosin light-chain phosphorylation [50]. Interestingly, male and female worms may use different pathways for cell communication with focal adhesion and gap junctions for males and tight junctions for females (figure 1).

Although GO and KEGG pathway analyses provided a clearer picture of molecular functions and pathways associated with GA genes, many GA genes have no known functions. Therefore, more extensive functional genomic data will be required to better understand the relation of these genes to the reproductive biology. These genes might play unique roles in reproductive processes in filarial nematodes, and characterization of these novel proteins would expand our knowledge of the molecules involved in parasite reproduction.

### Filarial gender-associated genes are most likely to have potential *C. elegans* homologues that are germline-, spermatogenesis-, oogenesis-, and embryogenesis-enriched

A number of key biological processes are conserved between the free living nematode *C. elegans* and parasitic nematodes [51], [52]. Data from *C. elegans* are often employed in a comparative manner to provide clues about potential roles of gender-associated genes in parasitic nematodes. Although *C. elegans* reproductive biology differs from parasitic nematodes, the adult hermaphrodite is functioning as a “conventional” female after terminating the production of sperm during the fourth larval stage [53]. Gender-associated genes identified in this study were compared *in silico* with *C. elegans* homologues for reduction-of-function phenotypes by RNAi [28,29,30,54,55 and 56]. The potential *C. elegans* homologues of gender-associated filarial genes tended to have a higher frequency of severe RNAi phenotypes such as embryonic lethal and sterility than other *C. elegans* genes. In *C. elegans*, approximately 13% of assayed genes have visible phenotypes with almost 10% showing either an embryonic lethal or sterile phenotype [57]. Fifty eight percent of the potential *C. elegans* homologues of filarial female-associated genes (501/850) and 34% of the potential *C.elegans* homologues of male-associated genes (211/605) have visible phenotypes such as maternal, embryonic and post-embryonic (Table S6). These findings are consistent with results described for other parasitic nematodes [5], [58]. We cross-referenced filarial GA gene expression profiles with global expression patterns in *C. elegans* (Table S7) reported by Reinke et al, who performed a genome-wide analysis of gene expression in *C. elegans* to identify germline- and sex-regulated genes [6]. The germline-enriched genes were classified further into specific expression patterns such as spermatogenesis-, oogenesis-, and intrinsic-enriched as shown in Table 4. Interestingly, 20% of the potential *C. elegans* homologues of filarial male-associated (119/605) and 18% of the potential *C. elegans* homologues of filarial female-associated elements (151/850) were annotated as “germline-enriched” in hermaphrodites producing both sperms and oocytes. In contrast, only 11% of all filarial elements on the BmV2 array (1,097/9,824) have potential *C. elegans* homologues annotated as “germline-enriched”. Thus, gender-associated filarial genes are more likely than other filarial genes to have potential homologues classified as “germline- enriched” in *C. elegans* hermaphrodites. In addition, sub-classification of expression patterns of filarial gender-associated genes and their potential *C. elegans* germline-enriched homologues are very similar (the oogenesis-enriched-Table S8; intrinsic-enriched Table S9; and spermatogenesis-enriched –Table S10). For example, most potential *C. elegans* germline-enriched homologues of filarial male-associated elements were annotated as “spermatogenesis-enriched” (73%, 87/119). By contrast, only a few potential *C. elegans* homologues of filarial female-associated elements (5%, 6/151) were annotated as “spermatogenesis-enriched” (Table 4). Genes with high expression during spermatogenesis are probably involved in spermatocyte differentiation [57]. One of these genes (BMC07594), which encodes a PIWI domain (P-element-induced Wimpy testis) containing protein that is thought to play a role in gene regulation in male gametes [41], [59], is highly expressed in filarial males (49 fold). In addition, the filarial male-associated list contains many genes with potential C. *elegans* homologues (106) that are expressed in hermaphrodites producing sperms (group B) and also in males (89) (group D) (Table 4). Similarly, the filarial female-associated gene list contains many genes with potential C. *elegans* homologues (75%, 113/151) that are expressed in hermaphrodites producing oocytes (Group C). The similar global gene expression patterns observed between the filarial gender-associated genes and their potential *C. elegans* homologues suggest that the biological functions of these genes may be conserved in reproductive processes [23].

**Table 4:** Expression patterns of *C. elegans* homologues for filarial gender-associated genes.

Expression patterns of *C. elegans* gene subset	*C. elegans* homologues for	*C elegans* homologues for
	male-associated elements (n = 605)	female-associated elements (n = 850)
**A: Germline-enriched transcripts in hermaphrodites**	**119**	**151**
** producing both oocyte and sperm**		
Intrinsic	16	72
oogenesis-enriched	16	73
spermatogenesis-enriched	87	6
**B: Spermatogenesis in hermaphrodites**	**106**	**9**
** producing sperm only**		
spermatogenesis-enriched	87	6
mixed spermatogenesis/somatic	19	3
**C: oogenesis in hermaphrodites**	**22**	**113**
** producing oocyte only**		
mixed oogenesis/somatic	6	40
oogenesis-enriched	16	73
**D: Germline-enriched transcripts in males**	**89**	**16**
shared spermatogenesis, germline -enriched	79	6
male sperm	5	0
shared germline	3	10
male germline	2	0

**Figure 2:**
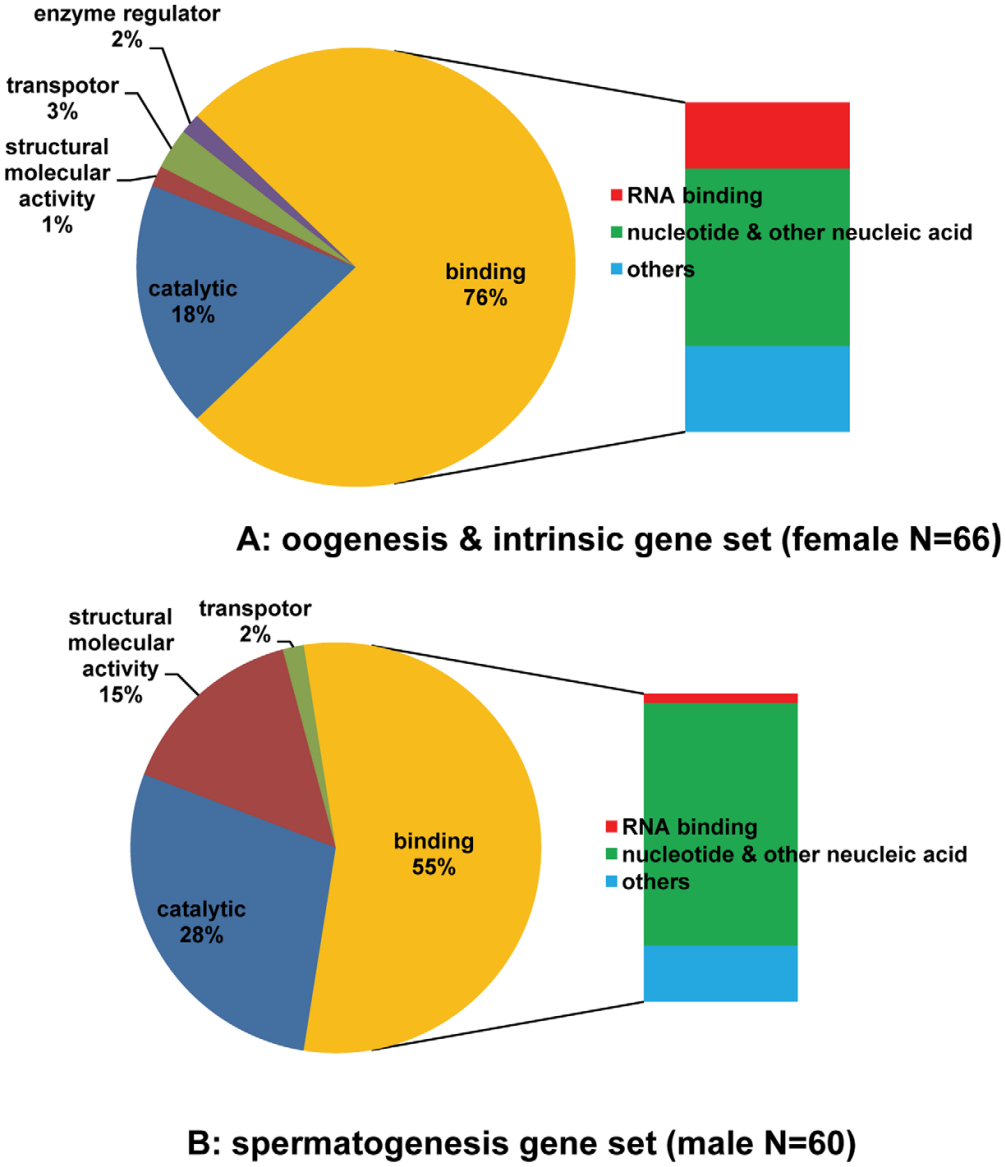
Functional categories of filarial gender-associated genes with specific expression pattern of potential *C. elegans* homologues. Pie charts show functional annotation of each of the major gene sets based on the assigned molecular function using gene ontology (GO) annotation. A: Functional categories of the female-associated genes with potential *C. elegans* homologues within oogenesis and intrinsic gene set. B: Functional categories of the male-associated genes with potential *C. elegans* homologues within spermatogenesis gene set. The sidebars divide genes encoding binding proteins into three categories: RNA binding, nucleotide and other nucleic acid and other bindings.

GO analysis provided clues regarding molecular functions of the filarial genes with potential *C. elegans* homologues in the intrinsic, oogenesis and spermatogenesis gene sets. For example, approximately 32% of all male- and 36% of all female-associated elements were associated with the “Molecular Function” GO. In contrast, 46% (66/145) of the elements in oogenesis and intrinsic combined and 69% (60/87) of the elements in the spermatogenesis subset were associated with “Molecular Function” GO terms (data not shown). Obviously, the percentage of the elements with “Molecular Function” in these subsets is much higher than that in the whole filarial gender-associated gene set. Most of the elements in these subsets encode predicted binding and catalytic proteins (83% of the elements in spermatogenesis and 94% of elements in oogenesis and intrinsic combined) (Figure 2). A hypothesis to explain the functional overlap among these sets may be that most events in germ cell development are similar in male and female worms (e.g., mitotic proliferation, recombination, and chromosome segregation). One striking difference between the predicted proteins encoded by the elements in spermatogenesis and oogenesis is in binding activity. RNA-binding activity is much higher in oogenesis and intrinsic sets than in the spermatogenesis set (Figure 2). Instead, the spermatogenesis set has more molecules involved in nucleotide binding (e.g., ATP binding). These results are consistent with those reported for *C. elegans* [6].

Embryogenesis is obviously an essential reproductive process. *C. elegans* embryogenesis is a powerful *in vivo* model for studying genes involved in embryo development, because its germline is highly sensitive to RNAi. Sonnichsen et al identified a set of 661 genes required for early embryogenesis in *C. elegans* [18]. Many of these genes are required for mitotic cell division in metazoans and are highly conserved across species [18], [ 28], [ 54], [ 55], [ and 60]. We found that 98 filarial gender-associated elements had potential *C. elegans* homologues expressed in early embryogenesis, and 80% of these (79/98) were female-associated (Table S11). Several of the filarial female-associated genes in this group encode proteins involved in protein synthesis, cell division and regulation of transcription (e.g., ribosomal protein large subunit family member rpl-27 and rpl-22, 40S ribosomal protein s27, cell division control protein 2, and histone family member (his-35). Male-associated genes with *C. elegans* homologues highly expressed in embryogenesis include actin, alpha tubulin and beta tubulin. A significant proportion of the embryogenesis associated genes encode hypothetical proteins (41/98). Additional work will be needed to assign functions to these proteins.

Genes identified in early embryogenesis are involved in various biochemical pathways such as cell cycle progression and DNA replication. One of these genes is the female-associated gene 12575.m00210, whose product is related to cell division control protein 2, which is highly expressed in early embryogenesis in *C. elegans*. Knockouts of these genes by RNAi in *C. elegans* cause serious phenotypes such as sterility and loss of osmotic integrity [18]. These genes are potential targets for drugs that could broadly interfere with embryogenesis in nematodes.

### *In situ* localization of gender-associated genes is consistent with their predicted molecular functions

**Figure 3:**
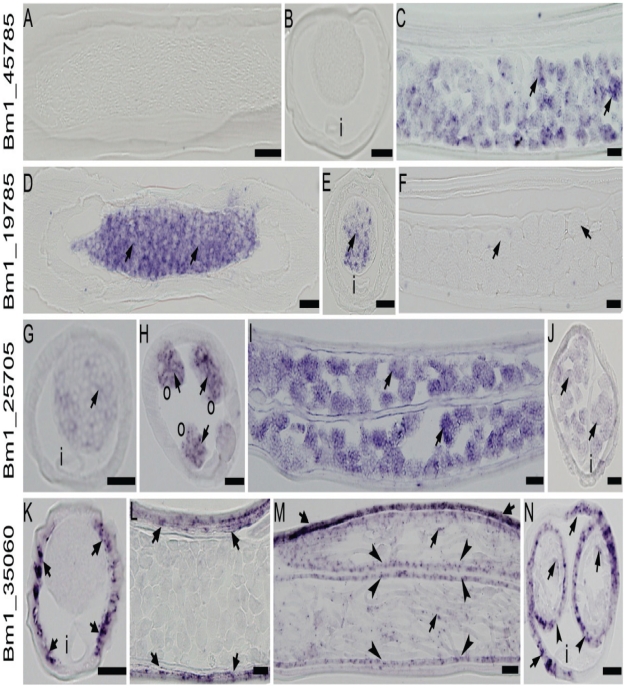
Expression patterns for four genes as revealed by *in situ* hybridization. Bm1_45785 (A–C)- female-associated; Bm1_19785 (D–F)-male-associated; Bm1_25705 (G–J)-female-associated and Bm1_35060 (K–N)-male-associated in adult *B. malayi*. A–C: hybridization signals were detected in morula stage embryos (C, arrows) in females with no signals in males (A,B); D–F: expression was detected in spermatocytes (D, arrows) and spermatids (E, arrow) in males, but no signal was detected in females (F); G–J: hybridization signals were detected in spermatocytes (G, arrow) in males and signals were also detected in developing oocytes (H, arrows) and morula stages embryos (I,J, arrows) in females; K–N: expression signals were present in muscle in males (K, arrows) and females (L,M,N, arrows); signals were also seen in stretched microfilariae (M,N, arrows) and in uterus epithelial cells (M,N, arrow heads) where stretched microfilariae were present. I: intestine; O: ovary. Scale bars: 20 µm.

Functional genomic approaches, such as RNAi, provide supportive information to better understand reproductive and developmental processes in parasites. However, there are significant technical challenges to this approach for *B. malayi* because of low efficacy and reproducibility [61]. Therefore, we have used ISH as an alternative approach to understand functions of gender-associated genes in reproductive processes. In *C. elegans*, genes with high expression during oogenesis encode proteins required for oocyte differentiation and development of the early embryo, and genes with high expression during spermatogenesis are most likely involved in spermatocyte specification and differentiation [57]. The *in situ* expression patterns of filarial gender-associated genes that have potential *C. elegans* homologues expressed either in oogenesis or spermatogenesis indicate that is also true in the *Brugia* parasites. For example, the female-associated gene BMC02383 (Bm1_36280), which has a potential *C. elegans* homologue in the germline subset that encodes caveolin-1, was exclusively expressed in embryos of *B. malayi* females as we previously reported [38]. In the current study, the same *in situ* pattern was observed as shown in Fig. 3 A–C for female-associated gene Bm1_45785, which has a potential *C. elegans* homologue annotated as a “germline- and oogenesis”-enriched gene and encodes a protein with a RNA recognition motif. In contrast, male-associated gene Bm1_19785 which encodes a novel protein is exclusively expressed in spermatocytes and spermatids in males as shown in Fig. 3 D–F. Interestingly, some of the gender-associated genes with predicted functions in reproductive processes, such as germline, oogenesis and embryogenesis are expressed in oocytes and embryos in females and in spermatocytes of males as previously reported [38]. As a new example, a female upregulated gene Bm1_25705 which encodes a putative peptidyl-prolyl cis-trans isomerase 11 is expressed in oocytes and morula stage embryos in females and in spermatocytes in males (Fig. 3 G–J). The finding that female-associated genes were expressed in males or vice versa by *in situ* is not surprising as gender differentially expressed genes may be either exclusively expressed in one sex (such as the female specific gene caveolin-1(BMC02383) and the male specific gene Bm1_19785) or differentially expressed but present in both sexes (female-associated gene Bm1_25705 (Fig. 3 G–J) and male-associated gene Bm1_35060 which encodes a troponin protein (Fig. 3 K–N)).

### Gender-associated genes encode excreted/secreted proteins (ES) and immunomodulatory molecules

ES products of filarial nematodes contain immunomodulatory molecules associated with immunological down-regulation in mammalian hosts [62], [ 63], [ and 64]. Several recent papers have identified major components in filarial ES products [20], [ 22], [ and 64]. We found that approximately 20% of the proteins encoded by the gender-associated elements were present in the secretomes of filarial nematodes (data not shown). Comparison of the Prorated Query Count percentage values of ES proteins (NQPCT), which is a measure of the relative abundance of a protein in a sample [22], [65], with the fold changes of corresponding gender-associated genes by microarray analysis showed a general agreement between the two types of data (Table 5). For instance, many gender-specific or abundant ES proteins were found to be encoded by gender-associated genes. However, there are exceptions. For example, two male-specific ES proteins were encoded by transcripts enriched in females, and vice versa. This suggests that the post-translational modification and/or secretory pathways might be gender specific in filarial nematodes. These genes are good candidates for future work to expand on our knowledge of post-translational modification and molecular biology of parasite secretions.

**Table 5:** Comparison between transcript levels and ES protein abundance of gender-associated genes.

		* Fold changes in		**ES protein abundance in		
Gene ID	Gene product name	M	F	MF	F	M
AI083432	Cuticular glutathione peroxidase	2		0.29	2.74	7.69
Bm1_07780	Immunogenic protein 3, putative	3		3.91	0.98	1.32
Bm1_06445	Transthyretin-like family protein	4		0.29	0.2	0.37
Bm1_04380	Transthyretin-like family protein	3		0.14	0.17	0.51
Bm1_07275	Core-2/I-Branching enzyme family protein	91		0	0.1	5.68
Bm1_04870	Putative uncharacterized protein	158		0	0.1	3.18
Bm1_13600	Major sperm protein 2, putative cytoskeletal MSP	153		0	0.13	1.74
Bm1_46740	Galactoside-binding lectin family protein	3		0	0.1	1.24
Bm1_41425	BM20, putative	2		0	0.54	0.66
Bm1_35885	MFP2, putative	10		0	0.03	0.88
Bm1_20065	Transthyretin-like family protein	4		0	0.03	0.29
Bm1_18805	Papain family cysteine protease containing protein,	2		0	0.1	0.15
Bm1_13900	Putative uncharacterized protein	2		0	0.03	0.15
Bm1_35600	ML domain containing protein	2		0.29	0	0.15
Bm1_29545	Zinc finger, C2H2 type family protein	2		1.16	0	0
Bm1_14040	Vespid allergen antigen homolog	3		1.16	0	0
Bm1_00290	Zinc finger, C2H2 type family protein	2		0.23	0	0
Bm1_20115	Putative uncharacterized protein	2		0	0.03	0
Bm1_30715	Putative uncharacterized protein	3		0	0.03	0
Bm1_23480	24 kDa secreted protein	4		0	0	0.81
Bm1_45510	MFP3, putative	5		0	0	0.51
Bm1_35295	Immunoglobulin I-set domain containing protein	2		0	0	0.44
Bm1_16920	Major sperm protein 3, putative	25		0	0	0.37
Bm1_09940	P40, putative	7		0	0	0.29
Bm1_55755	Major sperm protein, putative	41		0	0	0.29
Bm1_43360	Putative uncharacterized protein	90		0	0	0.15
Bm1_14035	SCP-like extracellular protein	3		0	0	0.07
Bm1_53295	Putative uncharacterized protein	43		0	0	0.07
Bm1_53680	Putative uncharacterized protein	5		0	0	0.07
TC3175	Superoxide dismutase	3.2		1.74	0.58	1.17
Bm1_37390	20S proteasome alpha5 subunit, putative		2	0	0.03	0
Bm1_28435	Bm-MIF-1, identical		2	0.72	8.91	0.15
Bm1_55850	Cyclophilin-type peptidyl-prolyl cis-trans isomerase-2, Bmcyp-2		2	1.45	0.47	1.1
Bm1_10970	14-3-3-like protein 2, putative		5	0.72	0.3	1.41
Bm1_44840	Glutathione S-transferase, N-terminal domain containing protein		3	0.29	0.54	0.07
Bm1_28235	Cytochrome c type-1, putative		2	0.29	0.2	0.22
Bm1_43675	Heat shock 70 kDa protein, putative		2	0	1.05	0.95
Bm1_23190	Hypothetical 86.9 kDa protein C30C11.4 in chromosome III, putative		3	0	1.05	0.15
Bm1_40520	Lectin C-type domain containing protein		7	0	0.54	0.22
Bm1_17400	Trypsin inhibitor, putative		3	2.61	0	0
Bm1_17270	Fasciclin domain containing protein		4	1.45	0	0
Bm1_45470	Bromodomain containing protein		2	0.43	0	0
Bm1_08645	Putative uncharacterized protein		8	0	0.81	0
Bm1_06185	Papain family cysteine protease containing protein		7	0	0.34	0
Bm1_42865	Brugia malayi antigen, putative		3	0	0.17	0
Bm1_39250	Protein disulphide isomerase, putative		2	0	0.1	0
Bm1_42375	Heavy neurofilament protein, putative		11	0	0.1	0
Bm1_22360	DB module family protein		4	0	0.07	0
Bm1_30230	Hypothetical 19.4 kDa protein ZC395.10 in chromosome III, putative		6	0	0.03	0
Bm1_45370	Cullin family protein		2	0	0.03	0
Bm1_23315	Cahepsin L-like non-peptiedase homolog		3	0	0.03	0
Bm1_33050	Embryonic fatty acid-binding protein Bm-FAB-1 precursor		2	0	0.03	0
Bm1_16810	Actin, putative		3	0	0	1.24
Bm1_11450	Chaperonin homolog HSP60, mitochondrial, putative		3	0	0	0.15

*Transcript level changes of gender-associated genes in M (male) and F (female) by microarray analysis.

**ES protein abundance measured by NQPCT (Prorated Query Count percentage values) at different stage MF (microfilaria), M (male) and F (female) reported by Moreno et al, 2008.

We also found that putative immunomodulatory proteins present in ES are encoded by gender-associated genes. Prominent among these are several antioxidant proteins including superoxide dismutase [66], and cuticular glutathione peroxidase (gp29) [67], [68]. Additionally, host cytokine mimics such as a homologue of macrophage migration inhibitory factor (BmMIF-1, Bm1_28435) [69], and cyclophilins (Bm1_55850) [70] were gender-associated and secreted [20], [22]. In addition to these well documented immunomodulatory proteins encoded by the gender-aaociated genes, we also found that newly identified secretory proteins with immunomodulatory potential such as 6 members of the transthyretin-like protein (TLP) family and three closely related hypothetical proteins (Bm1_11505; Bm1_01245; Bm1_09845) [64] were more highly expressed in male worms.

### Expression of filarial gender-associated genes is affected by tetracycline

Kumar et al identified and prioritized a pool of 589 potential drug targets in *B. malayi* using an *in silico* rational drug target selection algorithm [23]. We found that approximately 15% of these potential drug targets are encoded by the gender-associated transcripts (Table S12). The actual numbers could be higher; as only gender-associated genes with Pub_Locus numbers in our data (72% of total gender-associated elements) are included in the analysis. It is noteworthy that 18% of the top 40 potential drug targets are female-associated, including the PAN domain containing protein encoded by gene Bm1_36170.

Tetracyclines affect embryogenesis in some filarial adult females because they kill endosymbiotic bacteria of the genus *Wolbachia* [71], [72]. We cross-referenced gender-associated genes with genes that were recently reported to be differentially expressed in *B. malayi* adult worms after tetracycline treatment [14]. The findings are very intriguing. Many genes with decreased expression after tetracycline treatment were genes with female-associated expression (Table 6). This was especially true for genes that may be linked to embryogenesis. For instance, female-associated *B. malayi* cuticular collagen genes such as alpha-1 collagen type IX (Bm1_56350) and other putative collagens (Bm1_00775 and Bm1_26670) were down regulated at 7 days post-treatment [14]. These genes are involved in cuticle synthesis which is important for microfilaria production as microfilariae are surrounded by a cuticle. The reduced expression of these genes may reflect interruption of normal embryogenesis in worms after tetracycline. As another example, the *B. malayi* gene cathepsin L-like precursor (CPL-4) (Bm1_20385) highly expressed in females (10 fold), was down-regulated at both 7 and 14 days post-treatment. The protein encoded by CPL-4 has been suggested to play an important role during embryogenesis and larval development in filarial nematodes [73], [74]. Interestingly, several male-associated genes whose proteins are involved in energy metabolism were also down-regulated after tetracycline treatment. These included *B. malayi* genes encoding ATP synthase F0 subunit 6 and NADH dehydrogenase subunit 4L. Given the prominent role of gender-associated genes in embryogenesis, it is not surprising that expression of many of these genes was suppressed by a drug that affects embryogenesis.

**Table 6:** Expression of gender-associated genes suppressed by tetracycline [Ghedin et al, 2009].

Female-associated genes		
Matching Pub-Locus	Description	Days Post Treatment
BM1_00865	Excretory/secretory protein Juv-p120 precursor, putative	14
BM1_04280	Hypothetical protein	14
BM1_44740	Oxidoreductase, short chain dehydrogenase/reductase family protein	14
BM1_42865	Brugia malayi antigen, putative	14
BM1_20385	Cathepsin L-like precursor (CPL-4), putative	14
BMC01633	Hypothetical protein	14
BMW01267.350	Exonuclease family protein	14
BMC01618	Unknown	14
BMC06790	Unknown	14
BM1_00775	Collagen, putative	7
BM1_26670	Collagen, putative	7
BM1_00775	Collagen, putative	7
BM1_56350	Alpha-1 collagen type IX, putative	7
BM1_20385	Cathepsin L-like precursor (CPL-4), putative	7
BMC02037	Alpha-1 collagen type IX	7
AA675760	Collagen	7
BMC05233	Unknown	7
BMBC_gene_64.1319	Maltose transmembrane transporter	7

Male-associated genes		
BM1_05255	Hypothetical protein	14
BM1_29545	Zinc finger, C2H2 type family protein	14
BMC04259	NADH dehydrogenase subunit 4L [*Brugia malayi*]	7
BMC01596	Dual specificity phosphatase, catalytic domain containing protein	7

In summary, genes that are differentially expressed in male and female filarial worms potentially encode proteins that are essential for many activities including metabolism, adaptation to the mammalian host, immune evasion and (especially) reproduction. Our prior study provided an initial look at this topic and identified gender-associated genes that encode proteins with important functions such as fatty acid binding (Bm-FAB-1), a member of cathepsin L-like cysteine proteases family (Bm-CPL- 4), members of major sperm protein family (Bm-MSPs) and a cuticular glutathione peroxidase [21], [62], [ 67], [68], [ and 74]. The current study has greatly expanded the list of gender-associated filarial genes. Our bioinformatics analysis was supported by *in situ* studies performed for some genes. We reasoned that our results would be more valuable if they could be related to other recent developments in filarial research (secretome, drug targets and effects of tetracycline). We also identified pathways and processes that are associated with gender-enriched genes. Many of these pathways and processes are involved in reproduction and are also important for adaptation to specialized environments. Collectively, this dataset contains a tremendous amount of information about genes that are gender-associated in filarial nematodes and about their likely importance for various biological processes. This has improved our understanding of the molecular biology of reproduction in filarial worms as well as other essential functions for these parasites. We believe that future work with this type of integrated systems biology approach will lead to important new tools for controlling filarial nematodes.

## Supporting Information

Table S1 **List of genes and primer sequences tested by qRT-PCR.**
(0.03 MB XLS)

Table S2 **List of male- and female-associated genes identified by microarray analyses.**
(0.63 MB XLS)

Table S3 **BLAST output against NCBI NR of filarial gender-associated genes.**
(0.44 MB XLS)

Table S4 **InterPro domains of filarial gender-associated genes.**
(0.34 MB XLS)

Table S5 **KEGG pathways of filarial gender-associated genes.**
(0.11 MB XLS)

Table S6 **RNAi phenotypes of the potential *C.elegans* homologs of filarial gender-associated genes.**
(0.17 MB XLS)

Table S7 **Germline-enriched potential *C. elegans* homologs of filarial gender-associated genes.**
(0.07 MB XLS)

Table S8 **Oogenesis-enriched potential *C. elegans* homologs of filarial gender-associated genes.**
(0.03 MB XLS)

Table S9 **Intrinsic-enriched potential *C. elegans* homologs of filarial gender-associated genes.**
(0.03 MB XLS)

Table S10 **Spermatogenesis-enriched potential *C. elegans* homologs of filarial gender-associated genes.**
(0.03 MB XLS)

Table S11 **Potential *C. elegans* homologs of filarial gender-associated genes expressed in early embryogenesis.**
(0.05 MB XLS)

Table S12 **Potential gene candidates for drug targets [Kumar, et al., 2007] with GA expression identified in the current study.**
(0.06 MB XLS)
